# Measurement Invariance of the GAD-7 and CESD-R-10 Among Adolescents in Canada

**DOI:** 10.1093/jpepsy/jsab119

**Published:** 2021-11-13

**Authors:** Isabella Romano, Mark A Ferro, Karen A Patte, Scott T Leatherdale

**Affiliations:** 1 School of Public Health Sciences, University of Waterloo, Waterloo, ON, Canada; 2 Department of Health Sciences, Brock University, St. Catharines, ON, Canada

**Keywords:** adolescents, anxiety, depression, measurement invariance

## Abstract

**Objective:**

The primary objective of our study was to assess measurement invariance (by grade and sex) of the 7-item Generalized Anxiety Disorder (GAD-7) and 10-item Center for Epidemiologic Studies Depression Revised (CESD-R-10) scales in a sample of adolescents in Canada. If measurement invariance was demonstrated, our secondary objective was to estimate differences in scale scores across these subgroups.

**Methods:**

We used data from 59,052 adolescents in Year 7 (2018–19) of the COMPASS school-based study. Measurement invariance was tested within a multigroup confirmatory factor analysis framework. Differences in scale scores were estimated using mixed linear regression which accounted for school-level clustering and adjusted for relevant confounders.

**Results:**

Both the GAD-7 and CESD-R-10 demonstrated strict measurement invariance by sex and grade in our sample. Mean scale scores were higher among adolescents in grade 12 compared to grade 9 (β_GAD-7_ = 0.91, *p* < .001; β_CESD-R-10_ = 0.99, *p* < .001) and among female adolescents compared to males (β_GAD-7_ = 3.36, *p* < .001; β_CESD-R-10_ = 2.58, *p* < .001).

**Conclusions:**

Findings support the validity of the GAD-7 and CESD-R-10 for screening risk of generalized anxiety and depression among Canadian adolescents, and further indicate that differences observed in scale scores across subgroups reflect actual differences in risk for generalized anxiety and major depression, respectively.

## Introduction 

Approximately 12% of individuals will experience a major depressive episode during their adolescence, and nearly one-third of adolescents are estimated to experience an anxiety disorder (Merikangas, 2010). Compared to males, female adolescents are disproportionately impacted by internalizing disorders including generalized anxiety and major depression (Rosenfield & Mouzon, 2013; Van Droogenbroeck et al., 2018), and report experiencing higher levels of psychological distress (Boak et al., 2018). Male youth, on the other hand, are generally more likely to experience externalizing disorders (Rosenfield & Mouzon, 2013). Older adolescent age is another risk factor contributing to differences in mental health (Boak et al., 2018; Kilburn et al., 2018), although trajectories are reportedly steeper among females than males (Fink et al., 2015; Van Droogenbroeck et al., 2018). To further understand sex- and age-related differences in adolescents’ mental health, it is necessary that screening tools are appropriate for drawing valid conclusions regarding risk in research and pediatric psychology practice. Establishing validity is also vital to support screening efforts in general and specialty pediatric clinics, where 50% of pediatric patients with physical illness may experience poor mental health (Butler et al., 2018) and upwards of 30% meet criteria for clinical psychiatric diagnoses such as generalized anxiety or major depression (Tegethoff et al., 2015).

The 7-item Generalized Anxiety Disorder (GAD-7 [Spitzer et al., 2006]) and the 10-item Center for Epidemiologic Studies Depression Revised (CESD-R-10 [Andresen et al., 1994]) scales represent two of the most widely used tools to screen for risk of depression and anxiety, respectively, within clinical and research contexts. Studies have reported use of these tools to screen for anxiety and depressive symptoms in pediatric health settings including among pediatric endocrinology (Benson et al., 2020; Moyer et al., 2019), neurology (Hellebrekers et al., 2019; Martinović et al., 2006), and organ transplant (McCormick et al., 2020) patients. The GAD-7 has demonstrated validity for use among general adult populations (Löwe et al., 2008; Hinz et al., 2017) and, while Radloff’s (1977) original 20-item CESD has been validated extensively, fewer studies have focused on Andresen et al.’s (1994) shortened CESD-R-10. Björgvinsson et al. (2013), for example, found the CESD-R-10 to have strong psychometric properties for screening depression severity in a clinical sample.

However, there have been a paucity of studies assessing the psychometric properties of these tools among child and adolescent populations, despite being frequently used in research and within pediatric health settings (including specialty care clinics) to screen for anxiety and depressive symptoms. Two studies have explored the psychometric properties of the GAD-7 for screening for adolescent anxiety in the general population; first among a large sample of adolescents in Finland (Tiirikainen et al., 2019), followed by Adjorlolo (2019) who assessed validity among adolescents in Ghana. Findings from both studies supported the reliability, factorial validity, and construct validity of the GAD-7 in this age group. Among U.S. adolescents diagnosed with generalized anxiety disorder, the GAD-7 further demonstrated sufficient specificity and sensitivity for detecting symptoms (Mossman et al., 2017). With regard to the measurement of depressive symptoms, Bradley et al. (2010) investigated the factorial validity of the CESD-R-10 among a small community-based sample of adolescents in Canada, and their findings supported its use as a screening tool for adolescents in the community. Similar findings have been noted among a smaller sample of adolescents in France (Cartierre et al. 2011).

Relating to psychometric validation, *measurement invariance* refers to a statistical property indicating whether a given scale measures the same latent construct across subgroups within a sample (Brown, 2015). Establishing measurement invariance is a prerequisite for making meaningful group-level comparisons (Brown, 2015), whereas measurement *non-*invariance is indicative of bias due to differences between subgroups in their interpretation of the items comprising a scale (van de Schoot et al., 2012). In the latter case, observed differences in scores may be artifactual and invalid. Non-invariance of tools used to screen for adolescent anxiety and depression can pose negative implications for pediatric psychology contexts; if scale items were to be interpreted differently by females and males, conclusions made might result in ill-informed screening efforts or mistargeted interventions.

Only a handful of studies have assessed measurement invariance of either the GAD-7 or the CESD-R-10 among pediatric samples, and none within a Canadian adolescent context. Only one previous study has confirmed invariance by sex of the GAD-7 among an adolescent sample (Adjorlolo, 2019), but findings from this Ghanaian sample may not generalize adequately to adolescents in Canada given observed discrepancies in the experience of mental disorders such as anxiety across cultures (Schreier et al., 2010). Existing evidence also supports full invariance of the CESD-R-10 by sex among French adolescents (Cartierre et al., 2011) and youth in sub-Saharan Africa (Kilburn et al., 2018). One previous version of the CESD-R-10 has been validated for use specifically among adolescents, albeit in the United States, and found no evidence of inequivalence for gender or sex within their sample (Haroz et al., 2014). No previous studies have examined measurement invariance of the GAD-7 or CESD-R-10 across age groups (e.g., secondary school grade) among adolescents.

### Objectives and Hypotheses

Using survey data from a large sample of adolescents across Canada, our primary objective was to test measurement invariance of the GAD-7 and CESD-R-10 by sex and grade. If measurement invariance was demonstrated, our secondary objective was to estimate the extent to which differences in GAD-7 and CESD-R-10 scores can be predicted by sex and grade among adolescents in Canada. In light of existing (albeit limited) available evidence, we hypothesized that the GAD-7 and CESD-R-10 would demonstrate full measurement invariance within our study sample of Canadian adolescents. In the presence of invariance, we further hypothesized that GAD-7 and CESD-R-10 scale scores would be higher among females and adolescents in older grades.

## Methods

### Study Design

We conducted this study using data from Year 7 (Y_7_[2018–19]) of the COMPASS study, a prospective cohort study (2012–21) of adolescents attending secondary schools across Alberta, British Columbia, Ontario, and Quebec, Canada (Leatherdale et al., 2014). Schools were purposefully recruited based on permitted use of active information, passive consent data collection protocols, which are shown to support robust reporting of mental health among adolescents (Thompson-Haile et al., 2013). All adolescents attending participating secondary schools are eligible to participate. The COMPASS questionnaire (Cq) is a paper-and-pencil-based survey designed to be completed once annually during class time. In Y_7_, 74,501 participants across 136 schools (*n*_AB_ = 8; *n*_BC_ = 15; *n*_ON_ = 51; *n*_QC_ = 52) participated in COMPASS. We received approval from our institution’s Office of Research Ethics and participating school boards. Additional details on the study design and methods are available in print (Leatherdale et al., 2014) and online (www.compass.uwaterloo.ca).

### Instrument

The COMPASS mental health module (MH-M; Patte et al., 2017a, b) included the GAD-7 (Spitzer et al., 2006) to measure symptoms of generalized anxiety. Within the past 2 weeks, participants were asked to indicate the number of days they felt the following symptoms: 1) *feeling nervous, anxious, or on edge*, 2) *not being able to stop or control worrying*, 3) *worrying too much about different things*, 4) *trouble relaxing*, 5) *being so restless that it’s hard to sit still*, 6) *becoming easily annoyed or irritable*, and 7) *feeling afraid as if something awful might happen*. Responses were scored on a 4-point Likert scale (0 = *not at all*, 1 = *several days*, 2 = *over half the days*, 3 = *nearly every day*) with sum scores ranging from 0 to 21 (higher scores indicating greater symptoms). Internal consistency in our sample was α = 0.91.

The CESD-R-10 (Zhang et al., 2012) was included on the Cq MH-M (Patte et al., 2017a, b) as a measure of depressive symptoms. On a 4-point Likert scale (0 = *none or less than 1 day*, 1 = *1–2 days*, 2 = *3–4 days*, 3 = *5–7 days*), participants were asked to indicate how often they experienced each of the following in the past week: 1) *I was bothered by things that usually don’t bother me*, 2) *I had trouble keeping my mind on what I was doing*, 3) *I felt depressed*, 4) *I felt that everything I did was an effort*, 5) *I felt hopeful about the future*, 6) *I felt fearful*, 7) *my sleep was restless*, 8) *I was happy*, 9) *I felt lonely*, and 10) *I could not get* “*going*.” Individual sum scores ranged from 0 to 30 with higher scores representing greater symptomology; items 5 and 8, which measure positive affect, are reverse-coded. Internal consistency across CESD-R-10 items within our study sample was α = 0.83.

Participants responded to the question, “are you male or female” on the Cq and selected their sex from *male* or *female* response options. Participants also indicated their school grade (9, 10, 11, 12) and age (in years), which were highly correlated (*r* = 0.85). We relied on grade for the current analyses given its relevance to our study sample and correlation with age. Participants were also asked to indicate their available weekly spending/saving money ($0, $1–20, $20–100, $100+). Participants reported their ethno-racial identity, categorized as racialized (Black, Asian, Indigenous [First Nations, Métis, Inuit], Latin American/Hispanic, and/or other) or non-racialized (White) to more accurately describe race and ethnicity as a social construct, rather than as biological fact (Ross et al., 2020).

### Procedure

#### School-Level Variation

To account for potential variability introduced by clustering within schools, we first calculated the intra-class correlation coefficient (ICC) as an indicator of shared variation in participants’ GAD-7 and CESD-R-10 sum scores. We measured the presence of within-school clustering for each measure (ICC_GAD-7_ = 0.036, ICC_CESD-R-10_ = 0.025) and adjusted for these effects within our analyses.

#### Measurement Invariance

Using a multigroup confirmatory factor analysis (CFA), our procedure for testing measurement invariance of the GAD-7 and CESD-R-10 incorporated four sequential steps requiring increasingly stringent equality constraints on between-group (i.e., males vs. females, grade 9 vs. 12) model parameters. First, we tested configural invariance to confirm consistency of the GAD-7 and CESD-R-10 factor structures between groups; no equality constraints were imposed on the configural model in this step (Byrne, 2012). Second, as a prerequisite for making valid group comparisons (Bollen, 1989), we established in the metric model whether factor loadings for each GAD-7 and CESD-R-10 item were equivalent between our groups. The third step verified whether mean item-level differences were fully explained by mean factor-level differences by testing for equivalent item intercepts in the scalar model (Brown, 2015). In the fourth step, a strict model was specified to test invariance across item residuals as a requisite for defensible item-level comparisons (Steinmetz, 2009). This four-step approach to systematically imposing constraints allowed us to identify which specific parameters contributed to model misfit and, in turn, to differences in interpretation (van de Schoot et al., 2012).

Criteria by which we established full measurement invariance were determined *a priori*. First was adequate model fit at each level of testing as indicated by at least two (Tompke et al., 2020; Tompke & Ferro, 2019) of the following: the comparative fit index (CFI), square root mean residual (SRMR), or root mean standard error of approximation (RMSEA). Established cut-points for these indices were CFI ≥0.950; SRMR ≤0.080; and RMSEA ≤0.080 (Brown, 2015). The second criterion required that changes in fit indices did not exceed cut-points in, again, at least two cases of the following: ΔCFI ≤−0.010, ΔSRMR ≥0.030, or ΔRMSEA ≥0.015 (Chen, 2007). Given our large sample size, we did not rely on χ^2^ goodness-of-fit and Δχ^2^ as indices of model fit (Cheung & Rensvold, 2002). We conducted measurement invariance tests by adolescents’ sex and grade in Mplus version 8.6 (Muthén & Muthén, 1998–2020) with categorical estimation (weighted least squares means and variance adjusted estimator).

#### Differences in Sum Scores

If measurement invariance was established in the GAD-7 and/or CESD-R-10, we tested for differences in sum scores across sex and grade using mixed linear regression. We reported β-estimates from our model output alongside 95% confidence intervals (CIs), adjusted for participants ’ ethno-racial identity, province, and weekly spending money (as a proxy for individual-level socioeconomic status and/or part-time employment). We used SAS version 9.4 (SAS Institute, 2016) and accounted for school-level clustering using a multilevel model in PROC MIXED.

## Results

### Sample Characteristics

There were 59,052 adolescents in our analytic sample, among whom half were female (49.8%). Roughly one-third of adolescents reported a racialized identity (4.1% Black, 12.1% Asian, 2.9% Latin American/Hispanic, 15% other or Mixed) while a majority were non-racialized (White). Participants ranged in age from 12 to 19 years (mean = 15.6 years, SD = 1.2 years). The mean sum scores were 6.5 (SD = 5.7, range: 0–21) for the GAD-7 and 9.1 (SD = 6.1, range: 0–30) for the CESD-R-10.

Descriptive statistics for the GAD-7 and CESD-R-10 measures are presented in Table  I. One-way ANOVA tests showed significant variation in both measures for sex and grade subgroups. *Post* *hoc* pairwise comparisons showed that females differed significantly from males in GAD-7 and CESD-R-10 mean scores at *p* <.0001. For grade, GAD-7 and CESD-R-10 mean scores were significantly different across subgroups at *p* <.0001 except for participants in Grades 10 and 11. Additional descriptive statistics are provided in Supplementary File A (Tables S1 and S2). 

**Table I.** Descriptive Comparisons of GAD-7 and CESD-R-10 Scores by Individual Characteristics, Among Y_7_ COMPASS (2018–19) Participants

		GAD-7^a^ score			CESD-R-10^b^ score		
Measure	*n* (%)	Mean (SD)	*F*	*p*	Mean (SD)	*F*	*p*
Sex							
Male	29,637 (50.2)	4.9 (5.1)	5,114.9	<.0001	7.7 (5.4)	2,899.6	<.0001
Female	29,415 (49.8)	8.2 (5.8)			10.5 (6.3)		
Grade							
9	17,018 (28.8)	6.0 (5.5)	113.4	<.0001	8.5 (6.0)	90.4	<.0001
10	17,020 (28.8)	6.5 (5.7)			9.1 (6.0)		
11	15,766 (26.7)	6.7 (5.7)			9.3 (6.1)		
12^c^	9,248 (15.7)	7.3 (6.0)			9.8 (6.1)		
Ethno-racial identity							
White	38,849 (65.9)	6.4 (5.6)	57.1	<.0001	8.8 (6.0)	88.7	<.0001
Black	2,418 (4.1)	5.9 (5.8)			9.2 (5.9)		
Asian	7,152 (12.1)	6.4 (5.3)			9.6 (5.6)		
Latin American/Hispanic	1,677 (2.9)	6.5 (5.7)			9.4 (6.0)		
Other^d^ or Mixed	8,820 (15.0)	7.4 (6.1)			10.2 (6.5)		
Weekly spending money							
$0 or ‘don’t know’	18,111 (30.7)	6.5 (5.7)	3.0	.0304	9.1 (6.7)	2.0	.1118
$1–20	13,279 (22.5)	6.6 (5.7)			9.2 (6.0)		
$21–100	14,480 (24.5)	6.5 (5.5)			9.0 (5.9)		
$100+	13,182 (22.3)	6.7 (5.9)			9.1 (6.1)		
Province							
Alberta	3,223 (5.5)	7.1 (6.1)	209.1	<.0001	9.6 (6.3)	119.9	<.0001
British Columbia	10,068 (17.0)	6.6 (5.7)			9.5 (5.8)		
Ontario	29,953 (50.7)	7.0 (6.0)			9.4 (6.2)		
Quebec	15,808 (26.8)	5.6 (4.9)			8.3 (5.8)		
Total sample	59,052 (100.0)	6.1 (5.6)			9.1 (6.1)		

*Note*. SD = standard deviation.

a Higher scores indicate greater self-reported symptoms of anxiety.

b Higher scores indicate greater self-reported symptoms of depression.

c Note there are fewer participants in this category as there is no Grade 12 in Quebec.

d Includes participants who identified as Indigenous (First Nations, Métis, Inuit) on the Cq.

### Measurement Invariance of the GAD-7 and CESD-R-10

The baseline CFA model demonstrated overall excellent fit for the GAD-7 (CFI = 0.980; SRMR = 0.026; RMSEA = 0.051 [90% CI 0.047–0.053]) in the total sample. The CESD-R-10 also demonstrated excellent fit (CFI = 0.984; SRMR = 0.028; RMSEA = 0.024 [90% CI 0.023–0.025]), once correlations were specified amongst residuals for items 3–5, 3–8, 3–9, 5–8, and 8–9. Supplementary File B (Table S3) presents fit statistics for all baseline models by subgroup.

Measurement invariance testing results of the GAD-7 and CESD-R-10 by sex and grade are shown in Table  II. The configural models for sex and grade demonstrated excellent fit in the GAD-7 (sex: CFI = 0.978; SRMR = 0.027; RMSEA = 0.069 [90% CI 0.067–0.070]; grade: CFI = 0.978; SRMR = 0.028; RMSEA = 0.098 [90% CI 0.096–0.101]) and the CESD-R-10 (sex: CFI = 0.979; SRMR = 0.029; RMSEA = 0.035 [90% CI 0.034–0.036]; grade: CFI = 0.975; SRMR = 0.034; RMSEA = 0.046 [90% CI 0.044–0.048]). Equality constraints imposed on the factor loadings (metric model) did not result in a substantially worse fit for either scale, nor did equality constraints placed on the item intercepts (scalar model) or residuals (strict model). Full measurement invariance of the GAD-7 and CESD-R-10 was demonstrated by both sex and grade. Standardized, item-by-item factor loadings are available by subgroup in Table  III.

**Table II.** Measurement Invariance of the GAD-7 and CESD-R-10 Scales by Sex and Grade Among Y_7_ COMPASS (2018–19) Participants

		χ^2^ (df)	CFI	SRMR	RMSEA (90% CI)	Δχ^2^ (df)	ΔCFI	ΔSRMR	ΔRMSEA
GAD-7	Sex^a^								
	Configural	4,756.7 (28)	0.978	0.027	0.069 (0.067–0.070)	–	–	–	–
	Metric	4,097.3 (34)	0.981	0.029	0.058 (0.056–0.059)	219.4 (6)	0.003	0.002	−0.011
	Scalar	3,120.6 (47)	0.986	0.029	0.043 (0.041–0.044)	119.6 (13)	0.005	0.000	−0.015
	Strict	5,094.5 (55)	0.976	0.058	0.051 (0.049–0.052)	1,879.3 (8)	−0.010	0.029	0.008
	Grade^b^								
	Configural	3,524.4 (28)	0.978	0.028	0.098 (0.096–0.101)	–	–	–	–
	Metric	3,027.6 (34)	0.981	0.028	0.083 (0.080–0.085)	49.6 (6)	0.003	0.000	−0.015
	Scalar	2,391.7 (47)	0.985	0.028	0.062 (0.060–0.064)	76.7 (13)	0.004	0.000	−0.021
	Strict	1,461.9 (55)	0.991	0.037	0.045 (0.043–0.046)	284.8 (8)	0.006	0.009	−0.017
CESD-R-10	Sex^a^								
	Configural	2,694.2 (60)	0.979	0.029	0.035 (0.034–0.036)	–	–	–	–
	Metric	2,449.8 (69)	0.981	0.038	0.031 (0.030–0.032)	547.0 (9)	0.002	0.009	−0.004
	Scalar	2,653.7 (88)	0.980	0.041	0.029 (0.028–0.030)	643.1 (19)	−0.001	0.003	−0.002
	Strict	5,803.3 (99)	0.955	0.059	0.040 (0.039–0.041)	2,296.2 (11)	−0.025	0.018	0.011
	Grade^b^								
	Configural	1,693.8 (60)	0.975	0.034	0.046 (0.044–0.048)	–	–	–	–
	Metric	1,416.8 (69)	0.980	0.037	0.039 (0.037–0.041)	227.6 (9)	0.005	0.003	−0.007
	Scalar	1,464.0 (88)	0.979	0.038	0.035 (0.033–0.037)	233.5 (19)	−0.001	0.001	−0.004
	Strict	1,672.2 (99)	0.976	0.043	0.035 (0.034–0.037)	344.9 (11)	−0.003	0.005	0.000

*Note.* CFI = comparative fit index; CI = confidence interval; *df* = degrees of freedom; RMSEA = root mean standard error of approximation; SRMR = square root mean residual. All *χ*^2^ values were *p *<* *.001.

a Male versus female.

b Grade 12 versus 9.

**Table III.** Item-Level GAD-7 and CESD-R-10 Factor Loadings (Standardized) by Sex and Grade Among Y_7_ COMPASS (2018–19) Participants

			Factor loading (SE)			
Scale		Item	Female	Male	Grade 9	Grade 12
GAD-7	1	Feeling nervous, anxious, or on edge	0.84 (0.00)	0.86 (0.00)	0.86 (0.00)	0.89 (0.00)
	2	Not being able to stop or control worrying	0.91 (0.00)	0.92 (0.00)	0.93 (0.00)	0.94 (0.00)
	3	Worrying too much about different things	0.88 (0.00)	0.89 (0.00)	0.89 (0.00)	0.92 (0.00)
	4	Trouble relaxing	0.81 (0.00)	0.84 (0.00)	0.84 (0.00)	0.87 (0.00)
	5	Being so restless that it’s hard to sit still	0.70 (0.01)	0.73 (0.01)	0.73 (0.01)	0.78 (0.01)
	6	Becoming easily annoyed or irritable	0.66 (0.01)	0.69 (0.00)	0.87 (0.01)	0.73 (0.01)
	7	Feeling afraid as if something awful might happen	0.76 (0.00)	0.79 (0.00)	0.77 (0.01)	0.81 (0.01)
CESD-R-10	1	I was bothered by things that usually don’t bother me	0.67 (0.01)	0.71 (0.00)	0.71 (0.01)	0.73 (0.01)
	2	I had trouble keeping my mind on what I was doing	0.69 (0.01)	0.73 (0.01)	0.74 (0.01)	0.76 (0.01)
	3	I felt depressed	0.79 (0.00)	0.82 (0.00)	0.80 (0.01)	0.72 (0.01)
	4	I felt that everything I did was an effort	0.58 (0.01)	0.62 (0.01)	0.55 (0.01)	0.57 (0.01)
	5	I felt hopeful about the future	−0.06 (0.01)	−0.07 (0.01)	−0.02 (0.01)	−0.03 (0.01)
	6	I felt fearful	0.70 (0.01)	0.74 (0.00)	0.73 (0.01)	0.75 (0.01)
	7	My sleep was restless	0.59 (0.01)	0.63 (0.00)	0.63 (0.01)	0.65 (0.01)
	8	I was happy	−0.30 (0.01)	−0.33 (0.01)	−0.26 (0.01)	−0.28 (0.01)
	9	I felt lonely	0.74 (0.00)	0.77 (0.00)	0.75 (0.01)	0.77 (0.01)
	10	I could not get “going”	0.75 (0.01)	0.78 (0.00)	0.78 (0.01)	0.79 (0.01)

### GAD-7 and CESD-R-10 Item Responses and Sum Scores

Adjusted likelihood of an average one-point increase in GAD-7 (Model I) and CESD-R-10 (Model II) sum scores, predicted by individual characteristics, are shown in Table  IV. Compared to males, females reported higher average GAD-7 (β = 3.36) and CESD-R-10 (β = 2.58) sum scores. GAD-7 and CESD-R-10 scores generally increased with increasing school grade; compared to participants in grade 9, the likelihood of a one-point increase in GAD-7/CESD-R-10 sum score was increasingly higher for those in grade 10 (β_GAD-7_ = 0.49; β_CESD-R-10_ = 0.55), grade 11 (β_GAD-7_ = 0.64; β_CESD-R-10_ = 0.75), and grade 12 (β_GAD-7_ = 0.91; β_CESD-R-10_ = 0.99).

**Table IV.** Mixed Linear Regression Model Predicting GAD-7 and CESD-R-10 Scores Among Y_7_ COMPASS (2018–19) Participants

	Model I—GAD-7		Model II—CESD-R-10	
Measure	Β	95% CL	Β	95% CL
Sex				
Male (*ref.*)	0.00	–	0.00	–
Female	3.36*	3.35–3.36	2.58*	2.85–2.85
Grade				
9 (*ref.*)	0.00	–	0.00	–
10	0.49*	0.47–0.48	0.55*	0.54–0.55
11	0.64*	0.63–0.64	0.75*	0.74–0.75
12	0.91*	0.90–0.91	0.99*	0.99–1.00

*Note.* CL = confidence limit; *ref.* = reference category; SE = standard error. All estimates are adjusted for race/ethnicity, province, and weekly spending money. Model I: Predicts the likelihood of a one-point increase in GAD-7 score. Model II: Predicts the likelihood of a one-point increase in CESD-R-10 score.

* *p *<* *.001.

## Discussion

Using data collected among a large sample of Canadian adolescents enrolled in the COMPASS study, we sought to assess measurement invariance of the GAD-7 and CESD-R-10 by sex and grade. Our findings indicate the presence of strict measurement invariance within the GAD-7 and CESD-R-10, confirming that these screening tools equivalently measure symptoms of generalized anxiety and major depression, respectively, across adolescent subgroups. While the GAD-7 and CESD-R-10 are commonly used in pediatric settings for research and clinical screening purposes, this study is the first to explicitly test and confirm measurement invariance of both screening tools among adolescents. Our findings provide robust evidence that further supports the validity of the GAD-7 and CESD-R-10 for use in pediatric populations.

We found the psychometric properties of the GAD-7 and CESD-R-10 items to be sufficiently generalizable to adolescents by sex or grade. Statistical differences observed in GAD-7 and CESD-R-10 scores across subgroups may therefore be representative of true differences in symptoms of anxiety and depression, respectively. We identified greater risk of generalized anxiety and major depression among females than males; this is consistent with evidence that female adolescents are disproportionately impacted by internalizing disorders than males (Van Droogenbroeck et al., 2018), and typically report higher levels of psychological distress more generally (Boak et al., 2018). While the causal factors underlying sex differences in adolescent psychopathology are not fully understood, they are likely multifactorial and mediated by a variety of factors including pubertal differences in timing of cognitive development (van Beek et al., 2012) and associated gender roles (Patel et al., 2007; Rosenfield & Mouzon, 2013).

Our results also indicated that symptoms of anxiety and depression appear to increase with secondary school grade, which is also consistent with existing estimates (Beesdo et al., 2009; Boak et al., 2018). Previous research indicates that older adolescents tend to report greater symptoms of psychopathology than their younger counterparts (Boak et al., 2018; Kilburn et al., 2018); the incidence of anxiety and depressive disorders is thought to increase with age (Perou et al., 2013) as symptoms become increasingly consistent with DSM-5 criteria for adults (Mullen, 2018). Given our sample of adolescents aged 12–19 years, these findings also indicate an ability to detect age-related differences in anxiety and depressive symptoms among adolescents in general. Future research should confirm whether observed statistical differences at the population level may also equate to differences by sex and age in clinical pediatric contexts.

While the GAD-7 and CESD-R-10 screening tools have been previously validated across a number of clinical and non-clinical populations, our findings fill an important gap within the existing literature where evidence among pediatric populations was limited. Although Adjorlolo (2019) found measurement invariance of the GAD-7 among Ghanaian adolescents, we are unaware of any similar investigations among Canadian adolescents. As anxiety is not experienced ubiquitously across cultural contexts (e.g., there are cultural effects of individualism versus collectivism on social anxiety [Schreier et al., 2010]), findings from other studies may not generalize adequately to Canadian or North American adolescents; establishing validity of the GAD-7 for use in our population remains important for adolescent anxiety research and clinical screening practices. This also applies to previous research that has established measurement invariance of the CESD-R-10 among adolescents in France (Cartierre et al., 2011) and Sub-Saharan Africa (Kilburn et al., 2018); cross-cultural differences in depressive disorders have been observed among pediatric samples (Stewart et al., 2012). Haroz et al. (2014) found a version of the CESD-R-10 to be invariant by gender and sex within their U.S. adolescent sample.

### Implications

This study provides practical and clinical implications by furthering the validity of the GAD-7 and CESD-R-10 as symptom screening tools among adolescents. Pediatric health researchers and clinicians relying on the GAD-7 and CESD-R-10 should note that these screening tools exhibited strict measurement invariance in our general Canadian adolescent sample—suggesting that anxiety and depressive symptoms can be equivalently measured across gender and grade with minimal risk of measurement bias. We encourage replication of our analyses using other child and adolescent samples to further investigate the psychometric properties, including measurement invariance, of the GAD-7 and CESD-R-10 for specific use in clinical pediatric settings.

### Strengths and Limitations

Our data—representing a robust sample of adolescents across Alberta, British Columbia, Ontario, and Quebec, Canada—constitute a primary strength of our study. COMPASS is the largest ongoing longitudinal cohort study of adolescent behavioral and mental health in Canada. Further strengths pertain to our robust analytic approach (i.e., full-information maximum likelihood and accounting for school-level clustering). Our study is not without limitations. First, it is possible that our large sample may partially account for differences identified in the GAD-7 and CESD-R-10. Though group differences we observed are statistically significant, further research is warranted to confirm whether they are in fact clinically significant. Second, we accounted for sex and not participants ’ self-identified gender, as these data were not collected. Evidence suggests non-binary and transgender youth report experiencing disproportionate levels of psychopathology compared to cis-gender youth (Veale et al., 2017), thus additional work is needed to examine measurement invariance of the GAD-7 and CESD-R-10 across gender identity groups. Additionally, recognizing there are other social, structural, and systemic factors (e.g., racialization, discrimination) which can influence adolescents’ mental health experiences, future research should consider measurement biases within these broader contexts. To this point, it is imperative that future research consider the heterogeneity across race and ethnicity in recognition that grouping participants together into “other” and/or “mixed” identity groups may mask differences and fail to inform screening and intervention efforts. Third, we note these self-reported data are not nationally representative and generalizability of our findings to all adolescents in Canada is thus limited. However, COMPASS relies on purposive sampling procedures that contribute to large sample sizes, and use of active-information passive-consent data collection protocols supports robust youth self-report research (Chartier et al., 2008). Fourth, it should be noted that the GAD-7 and CESD-R-10 were examined in specific due to their availability as existing measures in the COMPASS host study; our findings of invariance cannot be generalized to other measures of the same constructs. Additional evidence is needed to confirm measurement invariance of other tools used to screen for symptoms of anxiety and depression, as well as for other mental disorders frequently observed among youth (e.g., externalizing disorders). We recommend similar tests of invariance be conducted for other measures used in pediatric psychology. Finally, it is a potential limitation that five residuals needed to be correlated in the CESD-R-10 to observe excellent fit of the CFA model. Although, it should be noted that specifying correlations among residuals does not usually have any negative impact on model results (Cole et al., 2007).

## Conclusion

Psychometrically valid screening tools are important for informing pediatric mental health research and supporting screening efforts in general and specialty pediatric settings. This study was the first to examine measurement invariance of the GAD-7 and CESD-R-10 in a large sample of general adolescents in Canada. We confirmed that the GAD-7 and CESD-R-10 were fully invariant across sex and grade subgroups. Consistent with previous research, our sample indicated higher symptoms of anxiety and depression among female adolescents and those in older school grades.

## Funding 

The COMPASS study has been supported by a bridge grant from the CIHR Institute of Nutrition, Metabolism and Diabetes (INMD) through the “Obesity—Interventions to Prevent or Treat” priority funding awards (OOP-110788; awarded to S.T.L.), an operating grant from the CIHR Institute of Population and Public Health (IPPH) (MOP-114875; awarded to S.T.L.), a CIHR project grant (PJT-148562; awarded to S.T.L.), a CIHR bridge grant (PJT-149092; awarded to K.A.P./S.L.), a CIHR project grant (PJT-159693; awarded to K.A.P.), and by a research funding arrangement with Health Canada (#1617-HQ-000012; contract awarded to S.T.L.). The COMPASS-Quebec project additionally benefits from funding from the Ministère de la Santé et des Services sociaux of the province of Quebec and the Direction régionale de santé publique du CIUSSS de la Capitale-Nationale.

## Supplementary Data

Supplementary data can be found at: https://academic.oup.com/jpepsy.

*Conflict* *of interest*: None declared. 

## Supplementary Material

jsab119_Supplementary_Data
