# Supplementary material for: Measurement Invariance of the GAD-7 and CESD-R-10 Among Adolescents in Canada
Source: J Pediatr Psychol. 2021 Nov 13;47(5):585–94. doi: 10.1093/jpepsy/jsab119 (PMC9113328; doi:10.1093/jpepsy/jsab119)
Supplement: jsab119_Supplementary_Data [file jsab119_supplementary_data.zip › JPP (Supplemental File A).docx]

| **Table S1**  *Descriptive comparisons of study measures by sex among Y_7_ COMPASS (2018-19) participants* | | | | | |
| --- | --- | --- | --- | --- | --- |
|  | **Sex**, *n* (%) | | | *χ*^2^ (*df*) | *p* |
| Measure | Males | Females | |  |  |
| **Grade** | | | | | |
| 9 | 8 684 (29.3) | 8 334 (28.3) | | 10.9 (3) | 0.0121 |
| 10 | 8 403 (28.4) | 8 617 (29.3) | |  |  |
| 11 | 7 862 (26.5) | 7 904 (26.9) | |  |  |
| 12^a^ | 4 688 (15.8) | 4 560 (15.5) | |  |  |
| **Ethno-racial identity** | | | | | |
| White | 19 329 (65.4) | 19 520 (66.5) | | 47.1 (4) | <0.0001 |
| Black | 1 374 (5.7) | 1 044 (3.5) | |  |  |
| Asian | 3 571 (12.1) | 3 581 (12.2) | |  |  |
| Latin American/Hispanic | 865 (2.9) | 812 (2.8) | |  |  |
| Other^b^ or Mixed | 4 411 (14.9) | 4 409 (15.0) | |  |  |
| **Weekly spending money** |  | |  |  |  |
| $0 or ‘don’t know’ | 9 248 (31.2) | 8 863 (30.1) | | 147.3 (3) | <0.0001 |
| $1-20 | 6 536 (22.1) | 6 743 (23.0) | |  |  |
| $21-100 | 6 766 (22.8) | 7 714 (26.2) | |  |  |
| $100+ | 7 087 (23.9) | 6 095 (20.7) | |  |  |
| **Province** |  | |  |  |  |
| Alberta | 1 602 (5.4) | 1 621 (5.5) | | 30.5 | <0.0001 |
| British Columbia | 5 159 (17.4) | 4 909 (16.7) | |  |  |
| Ontario | 15 228 (51.4) | 14 725 (50.1) | |  |  |
| Quebec | 7 648 (25.8) | 8 160 (27.7) | |  |  |
| *Note.* SD = standard deviation. ^a^Note there are fewer students in this category as there is no Grade 12 in Quebec. ^b^Includes students who identified as Indigenous (First Nations, Métis, Inuit) on the Cq. | | | | | |

| **Table S2**  *Descriptive comparisons of study measures by grade among Y_7_ COMPASS (2018-19) participants* | | | | | | | | | | | | |
| --- | --- | --- | --- | --- | --- | --- | --- | --- | --- | --- | --- | --- |
|  | **Grade**, *n* (%) | | | | | | | | | | *χ*^2^ (*df*) | *p* |
| Measure | 9 | | 10 | | | 11 | | | 12 | |  |  |
| **Sex** | | | | | | | | | | | | |
| Male | 8 684 (51.0) | 8 403 (49.4) | | | 7 862 (49.9) | | | 4 688 (50.7) | | | 10.9 (3) | 0.0121 |
| Female | 8 334 (49.0) | 8 617 (50.6) | | | 7 904 (50.1) | | | 4,560 (49.3) | | |  |  |
| **Ethno-racial identity** | | | | | | | | | | | | |
| White | 11 628 (68.6) | 11 468 (67.5) | | | 10 497 (66.7) | | | 5 256 (56.9) | | | 689.7 (12) | <0.0001 |
| Black | 653 (3.9) | 726 (4.3) | | | 637 (4.0) | | | 402 (4.4) | | |  |  |
| Asian | 1 578 (9.3) | 1 849 (10.9) | | | 1 964 (12.5) | | | 1 761 (19.1) | | |  |  |
| Latin American/Hispanic | 446 (2.6) | 451 (2.6) | | | 472 (3.0) | | | 308 (3.3) | | |  |  |
| Other^a^ or Mixed | 2 647 (15.6) | 2 493 (14.7) | | | 2 178 (13.8) | | | 1 502 (16.3) | | |  |  |
| **Weekly spending money** |  | | |  | | |  | | |  |  |  |
| $0 or ‘don’t know’ | 6 588 (38.7) | 5 378 (31.6) | | | 3 951 (25.0) | | | 2 194 (23.7) | | | 4 165.8 (9) | <0.0001 |
| $1-20 | 5 224 (30.7) | 4 090 (24.0) | | | 2 626 (16.7) | | | 1 339 (14.5) | | |  |  |
| $21-100 | 3 433 (20.2) | 4 178 (24.6) | | | 4 355 (27.6) | | | 2 514 (27.2) | | |  |  |
| $100+ | 1 773 (10.4) | 3 374 (19.8) | | | 4 834 (30.7) | | | 3 201 (34.6) | | |  |  |
| **Province** |  | | |  | | |  | | |  |  |  |
| Alberta | 447 (2.6) | 1 042 (6.1) | | | 981 (6.2) | | | 753 (8.1) | | | 4 401.4 (9) | <0.0001 |
| British Columbia | 2 417 (14.2) | 2 630 (15.4) | | | 2 708 (17.2) | | | 2 313 (25.0) | | |  |  |
| Ontario | 8 483 (49.9) | 8 060 (47.4) | | | 7 228 (45.8) | | | 6 182 (66.0) | | |  |  |
| Quebec | 5 671 (33.3) | 5 288 (31.1) | | | 4 849 (30.8) | | | 0 (0.0)^b^ | | |  |  |
| *Note.* SD = standard deviation.  ^a^Includes students who identified as Indigenous (First Nations, Métis, Inuit) on the Cq.  ^a^There is no Grade 12 in Quebec. | | | | | | | | | | | | |
