# Supplementary material for: Measurement Invariance of the GAD-7 and CESD-R-10 Among Adolescents in Canada
Source: J Pediatr Psychol. 2021 Nov 13;47(5):585–94. doi: 10.1093/jpepsy/jsab119 (PMC9113328; doi:10.1093/jpepsy/jsab119)
Supplement: jsab119_Supplementary_Data [file jsab119_supplementary_data.zip › JPP (Supplemental File B).docx]

| **Table S3**  *Table of baseline model fits for the GAD-7 and CESD-R-10 scales by sex and grade among Y_7_ COMPASS (2018-19) participants* | | | | | |
| --- | --- | --- | --- | --- | --- |
| Scale/Sample | | *χ*^2^ (*df*) | CFI | SRMR | RMSEA (90% CI) |
| **GAD-7** | **Total sample** | 2 654.3 (14) | 0.980 | 0.026 | 0.051 (0.049, 0.053) |
|  | **Sex** |  |  |  |  |
|  | Males | 2 245.5 (14) | 0.983 | 0.027 | 0.067 (0.064, 0.069) |
|  | Females | 2 124.5 (14) | 0.972 | 0.028 | 0.065 (0.063, 0.067) |
|  | **Grade** |  |  |  |  |
|  | Grade 9 | 1 533.5 (14) | 0.977 | 0.028 | 0.080 (0.077, 0.084) |
|  | Grade 12 | 1 979.0 (14) | 0.980 | 0.027 | 0.125 (0.120, 0.129) |
| **CESD-R-10** | **Total sample** | 17 112.2 (35) | 0.780 | 0.076 | 0.083 (0.082, 0.084) |
|  | **Total sample^a^** | 1 259.1 (30) | 0.984 | 0.028 | 0.024 (0.023, 0.025) |
|  | **Sex** |  |  |  |  |
|  | Males | 1 031.2 (30) | 0.986 | 0.025 | 0.031 (0.029, 0.032) |
|  | Females | 1 363.3 (30) | 0.975 | 0.034 | 0.036 (0.034, 0.037) |
|  | **Grade** |  |  |  |  |
|  | Grade 9 | 778.4 (30) | 0.982 | 0.036 | 0.039 (0.036, 0.041) |
|  | Grade 12 | 987.8 (30) | 0.964 | 0.029 | 0.060 (0.057, 0.063) |
| *Note. df* = degrees of freedom; CFI = comparative fit index; SRMR = square root mean residual; RMSEA = root mean standard error of approximation; CI = confidence interval. All *χ*^2^ values were *p* < 0.001. ^a^Correlations were specified amongst residuals for the following CESD-R-10 items to improve model fit: 3-5, 3-8, 3-9, 5-8, 8-8. This modified structure was used in baseline models and tests of invariance. | | | | | |
